# The Data set for Patient Information Based Algorithm to Predict Mortality Cause by COVID-19

**DOI:** 10.1016/j.dib.2020.105619

**Published:** 2020-04-24

**Authors:** Jing Li, Lishi Wang, Sumin Guo, Ning Xie, Lan Yao, Yanhong Cao, Sara W. Day, Scott C. Howard, J. Carolyn Graff, Tianshu Gu, Jiafu Ji, Weikuan Gu, Dianjun Sun

**Affiliations:** a Department of Orthopedic Surgery and BME-Campbell Clinic, University of Tennessee Health Science Center, Memphis, Tennessee, 38163, USA.; b Department of Basic Medicine, Inner Mongolia Medical University, Inner Mongolia, 010110, P. R. China.; c Department of Oncology, Hebei Chest Hospital, Lung Cancer Control and Prevention Center of Hebei Province, Shijiazhuang, Hebei, 050041, P. R. China.; d College of Business, University of Louisville, Louisville, KY, 40292, USA.; e Health Outcomes and Policy Research, College of Graduate Health Sciences, University of Tennessee Health Science Center, Memphis, TN 38103, USA.; f Center for Endemic Disease Control, Chinese Center for Disease Control and Prevention, Harbin Medical University; Key Laboratory of Etiologic Epidemiology, Education Bureau of Heilongjiang Province & Ministry of Health (23618104), 157 Baojian Road, Harbin, Heilongjiang, 150081, P. R. China.; g College of Nursing, University of Tennessee Health Science Center, Memphis, TN 38105, USA.; h Department of Neurology, Beijing Tiantan Hospital, Capital Medical University, Beijing 100050, PR China.; i Beijing Cancer Hospital and Key Laboratory of Carcinogenesis and Translational Research, Department of Gastrointestinal Surgery, Peking University Cancer Hospital and Institute, Beijing 100142, P. R. China.; j Research Service, Memphis VA Medical Center, 1030 Jefferson Avenue, Memphis, TN, 38104, USA.

**Keywords:** PIBA, Coronavirus, COVID-19, Death Rate, Normal distribution, Estimation, Prediction

## Abstract

The data of COVID-19 disease in China and then in South Korea were collected daily from several different official websites. The collected data included 33 death cases in Wuhan city of Hubei province during early outbreak as well as confirmed cases and death toll in some specific regions, which were chosen as representatives from the perspective of the coronavirus outbreak in China. Data were copied and pasted onto Excel spreadsheets to perform data analysis.

A new methodology, Patient Information Based Algorithm (PIBA) [1], has been adapted to process the data and used to estimate the death rate of COVID-19 in real-time. Assumption is that the number of days from inpatients to death fall into a pattern of normal distribution and the scores in normal distribution can be obtained by observing 33 death cases and analysing the data [2]. We selected 5 scores in normal distribution of these durations as lagging days, which will be used in the following estimation of death rate. We calculated each death rate on accumulative confirmed cases with each lagging day from the current data and then weighted every death rate with its corresponding possibility to obtain the total death rate on each day. While the trendline of these death rate curves meet the curve of current ratio between accumulative death cases and confirmed cases at some points in the near future, we considered that these intersections are within the range of real death rates.

Six tables were presented to illustrate the PIBA method using data from China and South Korea. One figure on estimated rate of infection and patients in serious condition and retrospective estimation of initially occurring time of CORID-19 based on PIBA.

Specifications table

**Table:** 

Subject	Death rate estimation using normal distribution, of mean, standard deviations and formulas.
Specific subject area	The data estimation focuses on the early estimation of death rate of infectious diseases, in particular, the disease COVID-19 caused by 2019-nCoV.
Type of data	Tables, Figures
How data were acquired	Data were obtained from official websites of provincial and central government of public health commissions of PR China and South Korea.
Data format	Collected data are formatted on Excel spreadsheets for analysing.
Parameters for data collection	Data include the total number of patients, total number of deaths, daily numbers of new patients, daily number of new deaths, from starting data of official report to the presented time, e.g., March 22, 2020.
Description of data collection	Data were collected through the cyberlinke of each official websites and copied and pasted the desired data onto Excel spreadsheets.
Data source location	City/Town/Region: Hubei province, Heilongjiang province. Country: PR China, South Korea
Data accessibility	Raw data are from three official websites which are publically avaialbe. Health Emergency Office of the National Health Commission of the People's Republic of China at http://www.nhc.gov.cn/yjb/new_index.shtml. Hubei Province and Wuhan are from the Health Commission of Hubei Province at http://wjw.hubei.gov.cn/fbjd/dtyw/. Health Commission of Heilongjiang province at http://wsjkw.hlj.gov.cn/index.php/Home/Zwgk/all/typeid/42.
Related research article [1]	Lishi Wang, Jing Li, Sumin Guo, Ning Xie, Lan Yao, Yanhong Cao, Sara W. Day, Scott C. Howard, J. Carolyn Graff, Tianshu Gu, Jiafu Ji, Weikuan Gu, Dianjun Sun. Real-time Estimation and Prediction of Mortality Caused by COVID-19 with Patient Information Based Algorithm. Science of the Total Environment. 2020, MS# STOTEN-D-20-06264. in press.

## Value of the data

• These data provide the scientific community with a new methodology to estimate the death rate and then predict the death cases during an epidemic.
• Scientific researchers, CDC employees, government officers for disease control and management, and public population, will benefit from these data.
• These data will be very useful for the studies with the purpose either of disease control management or of related sources preparation to combat against an outbreak.
• Due to the limited amount of data samples collected in this article, some factors, such as the phases of an outbreak and the measurements issued by the department of disease control that might impact the death rate of an epidemic, could be taken into for further insights and development of experiments with a large amount of data.

## 1 Data description

The data of 33 death cases in Table 1 have been collected from the official website of the Health Commission of Hubei Province in China, which include the date that patients have onset of symptoms, the date that patients began to be taken into ICU and the date of decease. With these data, the days both from symptoms appearance to death and from ICU intake to death can be calculated. Following normal distribution, the mean score μ and standard deviation σ can be calculated either. Thus the 5 selected scores (μ, μ ± σ and μ ± 2σ) in normal distribution can be obtain as the basic elements for the following estimation and prediction of death rate, which are respectively 2, 8, 13, 19, 25 days.

**Table 1:** 33 death cases in Wuhan city of Hubei province in China.

Patient No.	Gender	age	symptoms appearance	ICU intake	decease	days from symptoms appearance to death	days from ICU intake to death
1	M	70	1/16/2020	1/19/2020	1/23/2020	7	4
2	F	76		1/18/2020	1/24/2020	-	6
3	M	72	1/12/2020	1/18/2020	1/23/2020	11	5
4	M	79	1/12/2020	1/17/2020	1/24/2020	12	7
5	M	55	1/9/2020	1/19/2020	1/24/2020	15	5
6	M	87	1/13/2020	1/19/2020	1/23/2020	10	4
7	F	66	1/10/2020	1/19/2020	1/21/2020	11	2
8	M	58	1/4/2020	1/18/2020	1/24/2020	20	6
9	M	66		1/11/2020	1/21/2020	-	10
10	M	78	1/14/2020	1/23/2020	1/24/2020	10	1
11	M	65	1/13/2020	1/16/2020	1/23/2020	10	7
12	M	67	1/11/2020	1/15/2020	1/24/2020	13	9
13	M	58	12/24/2019	1/1/2020	1/23/2020	30	22
14	F	67	1/6/2020	1/12/2020	1/23/2020	17	11
15	F	82	1/11/2020	1/17/2020	1/23/2020	12	6
16	F	69		1/14/2020	1/22/2020	-	8
17	M	36	1/7/2020	1/9/2020	1/23/2020	16	14
18	M	73	12/29/2019	1/5/2020	1/22/2020	24	17
19	F	70	1/16/2020	1/18/2020	1/23/2020	7	5
20	M	81	1/10/2020	1/13/2020	1/21/2020	11	8
21	F	65	1/13/2020	1/15/2020	1/23/2020	10	8
22	F	70		1/13/2020	1/21/2020	-	8
23	M	53	1/10/2020	1/20/2020	1/21/2020	11	1
24	M	86	1/9/2020	1/9/2020	1/21/2020	12	12
25	M	65		1/11/2020	1/21/2020	-	10
26	M	84	1/7/2020	1/10/2020	1/22/2020	15	12
27	M	81		1/18/2020	1/22/2020		4
28	F	80	1/11/2020	1/18/2020	1/22/2020	11	4
29	F	82	1/12/2020	1/20/2020	1/22/2020	10	2
30	M	66	1/11/2020	1/16/2020	1/20/2020	9	4
31	M	89	1/13/2020	1/18/2020	1/19/2020	6	1
32	M	69	12/31/2019	1/4/2020	1/15/2020	15	11
33	M	33	1/10/2020	1/12/2020	2/6/2020	27	25

**Total cases**						**27**	**33**
**Standard deviation**						**5.75**	**5.51**
**The Mean**						**13**	**8**

*Notes:* CHD-Coronary heart disease.

The disease information in Table 2 has been collected from the public media before we resume data analysing with the same method of death rate estimation and prediction in South Korea as in China [1]. We have collected accumulative confirmed cases and deaths and then new confirmed cases and new deaths in South Korea.

**Table 2:** Disease information in South Korea.

date	2020-03-15	2020-03-14	2020-03-13	2020-03-12	2020-03-11	2020-03-10	2020-03-09
Accumulative confirmed cases	8162	8086	7979	7689	7755	7513	7478
Accumulative Deaths	75	72	67	66	60	60	53
New confirmed cases	76	107	110	114	242	35	165
New deaths	3	5	1	6	0	7	3
**date**	**2020-03-08**	**2020-03-07**	**2020-03-06**	**2020-03-05**	**2020-03-04**	**2020-03-03**	**2020-03-02**
Accumulative confirmed cases	7313	7041	6593	6284	5621	5186	4335
Accumulative Deaths	50	48	43	42	35	32	28
New confirmed cases	272	448	309	663	435	851	599
New deaths	2	5	1	7	3	4	7
**date**	**2020-03-01**	**2020-02-29**	**2020-02-28**	**2020-02-27**	**2020-02-26**	**2020-02-25**	**2020-02-24**
Accumulative confirmed cases	3736	3150	2337	1766	1261	977	833
Accumulative Deaths	21	17	16	13	12	11	8
New confirmed cases	586	813	571	505	284	144	231
New deaths	4	1	3	1	1	3	2
**date**	**2020-02-23**	**2020-02-22**	**2020-02-21**	**2020-02-20**	**2020-02-19**	**2020-02-18**	**2020-02-17**
Accumulative confirmed cases	602	436	209	111	58	31	30
Accumulative Deaths	6	2	2	1	0	0	0
New confirmed cases	166	227	98	53	27	1	1
New deaths	4	0	1	1	0	0	0
**date**	**2020-02-16**	**2020-02-15**	**2020-02-14**	**2020-02-13**	**2020-02-12**	**2020-02-11**	**2020-02-10**
Accumulative confirmed cases	29	28	0				
Accumulative Deaths	0	0	0				
New confirmed cases	1	28	0				
New deaths	0	0	0				

According to the analysis result from Table 1, we have selected 5 scores (μ, μ ± σ and μ ± 2σ) in normal distribution which are respectively 2, 8, 13, 19, 25 days. When we calculate the death rate by dividing death cases with confirmed cases, these confirmed cases should be the ones on the 2^nd^, 8^th^, 13^th^, 19^th^ and 25^th^ day before the day of prediction.

Death rate 1 is calculated by dividing new death cases with new confirmed cases. Death rate 2 is calculated by dividing accumulative death cases with accumulative confirmed cases. When the death rate came out with a negative value or no value, that means the new confirmed cases might be wrong for some reason or there's no new cases on several days before. We corrected a negative death rate or no value into zero (in red), and then added the new death case to the one of the next days (in green).

Each score we selected in normal distribution has a specific possibility when we take them into consideration of representatives in bell curve [1]. When we weighted each death rate on a day with their corresponding possibilities and then sum, the total death rate on each day can be obtained.

Each curve consisting of several death rate will have a trendline and thus a formula to describe this trend as well as the current ratio between accumulative death cases and confirmed cases on each day (Table 4).

The current ratio between accumulative death cases and confirmed cases is calculated by dividing accumulative death cases with accumulative confirmed cases on each day.

The trendlines of death rate 1 and death rate 2 tend to intersect with the trendline of the current ratio finally, because the current ratio will be the real death rate at the end of epidemic. We considered that the intersection value of three trendline (death rate1 and 2, current ratio) will drop in the range of real death rate. When we calculated the death rate separately with the corresponding formula of their trendlines, two intersections have been acquired (Table 5-B). We pick the maximum value between them to predict new death cases in the following days (Table 6).

This table listed the number of deaths from March 16, 2020 to March 22, 2020 based on lagging days of 8, 13, and 19 days. The upper parts are predicted number of deaths based on days of 8, 13, and 19 days of the PIBA method. The lower part list the predicted minimum and maximum number of deaths, and actual reported deaths in each of the seven days.

**Fig. 1:**
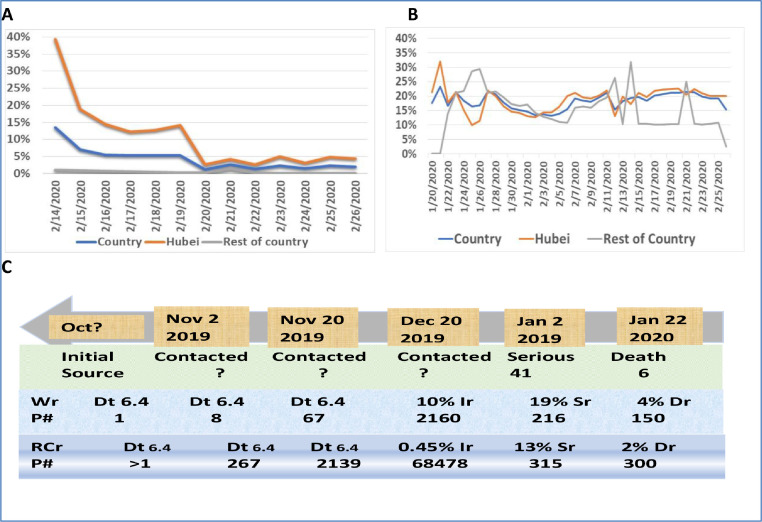
Estimated rate of infection and patients in serious condition and retrospective estimation of initially occurring time of CORID-19 based on PIBA data.

Fig. 1. Rates of 2019-nCoV infection and rate of patients in serious medical condition. Total rate in China (blue color), Hubei (orange color) and rest of country (grey color). Numbers on the vertical axis indicate the percentage of infections. Numbers on the horizontal axis indicate the date. Fig. 1A. The infection rates. Fig. 1B. The rate of patients in serious medical condition. Fig. 1C. Retrospective estimation of start time of disease based on PIBA and known information of patients in Wuhan. *Wr = Based on the rate of Wuhan; Rcr = Based on the rate from the rest of the country; Dt=doubling time [9]; Ir=infection rate; Sr= serious rate; Dr = death rate.

## 2 Experimental design, materials, and methods

Tables are produced based on the Patient Information Based Algorithm (PIBA) [1]. PIBA has been adapted when estimating the death rate of COVID-19 in Real-time with publicly posted data. Following normal distribution, the different durations with different possibilities between symptom appearance and death have been derived from analysing 33 death cases in Wuhan city of Hubei province in China [2]. Based on these results, the total death rate in regions can be calculated specifically by putting in the different death rates with different durations together. While the trendline of these death rate curves meet the curve of current ratio between accumulative death cases and confirmed cases at some points in the near future, we considered that these intersections are within the range of real death rates. The data analysis was all following normal distribution, either in calculating the possibility of every selected score or in estimating the death rate.

After collection of data of COVID patients from South Korea, the data was analysed with PIBA method as indicated above (Table 2). The death rate was first estimated (Table 3). The death rate then was calculated (Table 4). Following estimations, the PIBA method then was used to predict the number of deaths in the following week (Table 5). The predicated death numbers then were compared to the real death numbers (Table 6).

**Table 3:** Death rate estimation in South Korea.

3-A: Death rate analysis in South Korea					
Death rate 1 from the date Symptoms	2020-03-15	2020-03-14	2020-03-13	2020-03-12	2020-03-11
Mean-13	0.50%	0.85%	0.12%	1.05%	0.00%
1STDEV-8	0.67%	1.62%	0.15%	1.38%	0.00%
1STDEV-19	2.08%	2.16%	0.60%	2.64%	0.00%
2STDEV-25	11.11%	500.00%	100.00%	600.00%	0.00%
2STDEV-2	2.76%	0.00%	0.41%	17.14%	0.00%
**Death rate2 from the date Symptoms**	**2020-03-15**	**2020-03-14**	**2020-03-13**	**2020-03-12**	**2020-03-11**
Mean-13	1.73%	1.93%	2.13%	2.82%	3.40%
1STDEV-7	1.07%	1.09%	1.07%	1.17%	1.16%
1STDEV-19	7.68%	8.64%	11.13%	15.14%	28.71%
2STDEV-25	129.31%	232.26%	223.33%	227.59%	214.29%
2STDEV-1	0.94%	0.94%	0.86%	0.88%	0.80%


3-B: The total Death rate weighted by the possibilities of selected scores in normal distribution					
Date	2020-03-15	2020-03-14	2020-03-13	2020-03-12	2020-03-11
**Death rate 1 from the date Symptoms**	**1.79%**	**37.24%**	**6.96%**	**42.72%**	**0.00%**
Mean-13	0.19%	0.33%	0.05%	0.40%	0.00%
1STDEV-8	0.16%	0.39%	0.04%	0.33%	0.00%
1STDEV-19	0.50%	0.52%	0.15%	0.64%	0.00%
2STDEV-25	0.74%	33.50%	6.70%	40.20%	0.00%
2STDEV-2	0.18%	0.00%	0.03%	1.15%	0.00%
**Date**	**2020-03-15**	**2020-03-14**	**2020-03-13**	**2020-03-12**	**2020-03-11**
**Death rate 1 from the date Symptoms**	**11.50%**	**18.72%**	**18.79%**	**20.33%**	**22.94%**
Mean-13	0.66%	0.74%	0.81%	1.08%	1.30%
1STDEV-8	0.26%	0.26%	0.26%	0.28%	0.28%
1STDEV-19	1.86%	2.09%	2.69%	3.66%	6.95%
2STDEV-25	8.66%	15.56%	14.96%	15.25%	14.36%
2STDEV-2	0.06%	0.06%	0.06%	0.06%	0.05%

**Table 4:** Current ratio between accumulative death cases and confirmed cases.

Date	2020-03-15	2020-03-14	2020-03-13	2020-03-12	2020-03-11
Current ratio between accumulative death cases and confirmed cases	0.92%	0.89%	0.84%	0.86%	0.77%

**Table 5:** Death rate estimation in South Korea.

5-A: Death rate derived from the formula of trendlines						
Date	3/11/2020	3/12/2020	3/13/2020	3/14/2020	3/15/2020	3/16/2020
**Death rate 1**	3.95%	26.92%	30.57%	21.26%	5.35%	
**current ratio**	0.79%	0.82%	0.85%	0.88%	0.91%	0.94%
**Death rate 2**	23.35%	20.90%	18.45%	16.00%	13.55%	11.10%

Date	3/17/2020	3/18/2020	3/19/2020	3/20/2020	3/21/2020	

**Death rate 1**						
**current ratio**	0.97%	1.00%	1.03%	1.06%	1.09%	
**Death rate 2**	8.65%	6.20%	3.75%	1.30%		


**5-B**: The intersect points of three trendlines			
	intersect 1	intersect 2	**Max. value**
Death rate in South Korea	0.92%	1.06%	**1.06%**

**Table 6:** Deaths prediction by PIBA and actual death data in South Korea.

Date	3/22/2020	3/21/2020	3/20/2020	3/19/2020	3/18/2020	3/17/2020	3/16/2020
7 lagging day	1	1	1	3	0	2	3
13 lagging day	2	3	5	3	7	5	9
19 lagging day	9	6	6	9	6	5	3

Date	3/22/2020	3/21/2020	3/20/2020	3/19/2020	3/18/2020	3/17/2020	3/16/2020

Min	1	1	1	3	0	2	3
Max	9	6	6	9	7	5	9
Actual deaths	2	8	3	7	3	6	0

Fig. 1 is produced based on the following procedure. Up to February 25, 2020, the total accumulated number of infected patients in China is 78,064 (data only from mainland China). The number of new cases per day has not increased in the past 9 days. The total accumulated number of people who were in close contact with an infected person is 647,406. Thus, by simply dividing the number of infected persons by the number of contacted persons, the total infection rate is only 12%, considerably lower than expected. Prior expectation has been much higher, based on multiple infectious routes [3], [4]. Using our formula, the results indicate that the current infectious rate is even lower than the rate based on the total numbers (see Fig. 1A). The infectious rate in Hubei province is currently around 4%, although previously the rate was as high as 39%. On average, the infectious rate overall in China is about 4%, while in Hubei it is 10%. In the rest of the country, it is 0.46%. Among the inpatients, the rate in serious medical condition ranges from 10% to 30% (see Fig. 1B), while it averages at 18% in China, 19% in Hubei, and 13% in the rest of country (except Hubei). Based on the estimated death rate, on January 22, there should be a total of 150 to 300 inpatients (see Fig. 5C). Based on the rate of patients who are severely ill among all patients, on January 2, there should be 216 to 315 patients. Based on the effective infection rate and based on the assumption of one week or 14 days from close contact to the onset of symptoms, there might be 2,160 to 68,478 people who were infected around December 20, 2019. If we believe the epidemic doubling time is approximately 6 days, the initial infection source may date back to as early as November or October 2019.
